# Liquid Phase adsorption kinetics and equilibrium of toluene by novel modified-diatomite

**DOI:** 10.1186/s40201-014-0148-9

**Published:** 2014-12-12

**Authors:** Reza Khalighi Sheshdeh, Saeed Abbasizadeh, Mohammad Reza Khosravi Nikou, Khashayar Badii, Mohammad Sadegh Sharafi

**Affiliations:** School of Chemical Engineering, College of Engineering, University of Tehran, Tehran, Iran; Department of Chemical Engineering, Tarbiat Modares University, Tehran, Iran; Ahwaz Faculty of Petroleum, Petroleum University of Technology (PUT), Ahwaz, Iran; Department of Environmental Researches, Institute for Color Science and Technology (ICST), Tehran, Iran

**Keywords:** Diatomite, Adsorption, Toluene, Isotherm, Kinetics

## Abstract

The adsorption equilibria of toluene from aqueous solutions on natural and modified diatomite were examined at different operation parameters such as pH, contact time, initial toluene concentration was evaluated and optimum experimental conditions were identified. The surface area and morphology of the nanoparticles were characterized by SEM, BET, XRD, FTIR and EDX analysis. It was found that in order to obtain the highest possible removal of toluene, the experiments can be carried out at pH *6*, temperature *25°C*, an agitation speed of *200 rpm*, an initial toluene concentration of *150 mg/L*, a centrifugal rate of 4000 rpm, adsorbent dosage = *0.1 g* and a process time of *90 min*. The results of this work show that the maximum percentage removal of toluene from aqueous solution in the optimum conditions for NONMD was *96.91%* (*145.36 mg/g*). Furthermore, under same conditions, the maximum adsorption of natural diatomite was *71.45%* (*107.18 mg/g*). Both adsorption kinetic and isotherm experiments were carried out. The experimental data showed that the adsorption follows the Langmuir model and Freundlich model on natural and modified diatomite respectively. The kinetics results were found to conform well to pseudo-second order kinetics model with good correlation. Thus, this study demonstrated that the modified diatomite could be used as potential adsorbent for removal of toluene from aqueous solution.

## Introduction

Wastewater pollutants discharged from pharmaceutical and chemical industries contain various types of aromatic compounds, such as toluene, pyridine, and nitrobenzene, which are very harmful to human health and the ecosystem [1]. In the past decades, extensive attention has been paid to develop efficient and cost-effective methods for removal of these pollutants. Various technologies were used for the treatment of organic pollutants, including adsorption, biodegradation, chemical oxidation, photo-degradation, solvent extraction, membrane separation, etc. [2,3]. Among these approaches, adsorption is still the most versatile and widely used method because of its high efficiency and simple operation conditions. Various adsorbents, such as active carbon, coal, fly ash, modified clays, polymeric resins, metal oxides, silicas, and zeolites, were applied to adsorb aromatic compounds in aqueous solutions [4-7]. For example, Roostaei and Tezel investigated toluene or benzene adsorption on silica gel, activated alumina, activated carbon, and zeolites, and found that the latter two materials provide a higher adsorption capacity for phenol [8-10]. Rodriguez and co-workers described activated carbons as good adsorbents for toluene and benzene [11-13]. Magnetic porous carbon microspheres have been reported with good adsorption properties for toluene and benzene [14-16]. Toluene and benzene and often coexist in wastewater because they are widely used in many industrial products and processes. Due to their biological toxicity to microorganisms, traditional biotreatment cannot be applied for an economical and effective degradation. Additionally, their coexistence will lead to the loss of degradation ability of bacteria to other pollutants [17-19]. Therefore, it is necessary to separate benzene and toluene from wastewater prior to their discharge into water bodies. Toluene is a typical indoor pollutant and its discharge may produce irritation of the eyes and the respiratory tract, nausea, headache, fatigue, dullness and thirst, even at very low concentrations [4-8]. Toluene is well known for its neurotoxicity and exposure to it may decrease neuronal activities in vitro and cause mental depression and cognitive impairment in humans [1,6]. Toluene inhalation also results in various symptoms such as fatigue, headache, vertigo and ataxia. It is rapidly absorbed through respiratory and gastrointestinal tracts and, to a lesser extent, through the skin. American Conference of Governmental Industrial Hygienists (ACGIH) has recommended an *8-h* time-weighted average (TWA) of *50 ppm* (*189 mg/m*^*3*^) for toluene to protect against effects on the central nervous system. These studies also confirmed that porosity and surface oxygen group content have major influences on low-concentration-*VOC* adsorption [18-20]. Natural diatomite (*SiO*_*2.*_*nH*_*2*_*O*) is made up from the skeletons of aquatic plants called diatom that usually is a pale-coloured, soft, lightweight siliceous sedimentary rock [21]. Diatomite contains a wide variety of shape and sized diatoms, typically *10-200 μm*, in a structure including up to *80-90%* pore spaces [19-21]. Diatomite’s extremely porous structure, low density and high surface area make it suitable as an adsorbent for organic and inorganic chemicals [22,23]. In general, the literature includes few experimental data for modified natural adsorbent for removal of VOCs pollutants. In the present investigation, for the first time, the usage of Nickel Oxide Nanoparticles-Modified Diatomite (NONMD) for the adsorption of toluene has been studied, as a less expensive adsorbent for removal of toluene from an aqueous medium. The equilibrium and kinetic study are investigated to observe the effects of various process parameters such as pH, contact time, initial toluene concentration, calcinations and sorbent dosage on the adsorption process. Equilibrium data are attempted by various adsorption isotherms including Langmuir, Freundlich and Brunauer-Emmett-Teller (BET) isotherms in order to select an appropriate isotherm model. Moreover, a kinetics study of the adsorption process is also considered to describe the rate of sorption. Comparing natural and modified diatomite (NONMD) well shows that this process was successful and the removal percentage increased [24,25].

## Experimental procedure

### Preparation of adsorbent

Diatomite sample was obtained from Tabriz, Iran. The sample was washed several times with distilled water and *HCl* (*1 M*) to remove fines and other adhered impurities and to achieve neutralization. The sample was finally filtered, dried at 60°C for 24 h, and stored in closed containers for further use.

The nanoparticles of *NiO* were synthesized by using following reaction (Equation 1):

1 $$ N{i}_2S{O}_4+2 NaOH\to Ni{(OH)}_2+N{a}_2S{O}_4 $$

The nanoparticles of *NiO* were synthesized by adding *NiSO*_*4*_ and *NaOH* (*1 M*) to the solution. It means that *2.0 g* of previously dried diatomite was added to *25 ml* of Nickel hydroxide (*1 M*), Stirrer speed of *200 rpm*, for *1 h*. The new material, *Ni*-diatomite was sequentially separated by filtration. The calcination process was carried out by placing Modified diatomite sample in the furnace at *250°C* for *4.5 h*. The sample was then allowed to cool in a desiccator. The modified sample was used to examine the effect of nickel oxide nanoparticles, silanol groups and the role of pore size distribution on the adsorption process. FT-IR spectra and EDX analysis illustrate that the raise of metal oxide content at the modified diatomite can be the main reason for increasing the adsorption capacity. In industries, there are many heavy metals contaminated materials. Recovery of materials and clean up these contaminations is very difficult and expensive [26-34]. This investigation can be a useful method to change a poisonous industrial waste to a valuable by product.

### Materials and solutions

Toluene (AR grade min. *99.6%* Merck) was supplied by Quick Lab Sdn. Bhd., Ipoh, Perak. Distilled water was throughout employed as solvent. For adsorption experiments, various concentrations of toluene solutions (*100*, *150*, *200*, *250* and *300 mg/L*) were prepared. The pH measurements were made using Hach pH meter. These chemicals were purchased from Merck, Germany.

## Adsorption procedure

The adsorption experiments were performed by mixing various amounts of diatomite (*0.03 – 0.12 g*) in *100 mL* of toluene solutions with varying concentrations (ranging from *100-300 mg/L*) at natural pH (*pH = 6*). The natural pH to determine the maximum toluene removal could be achieved with diatomite, because this pH was more suitable for industrial purposes. Adsorption experiments were conducted at optimum amount of diatomite (*0.1 g*) at pH *6*, an agitation speed of *200 rpm* and temperature *25 ± 1°C* for *1 h* to attain equilibrium conditions. The changes of absorbance were determined at certain time intervals (*5*, *10*, *30*, *60* and *90 min*) during the adsorption process. After adsorption experiments, the toluene solutions were centrifuged for 10 min in a Hettich EBA20 centrifuge at *4000 rpm* in order to separate the sorbent from the solution and toluene concentration was then determined. The amount of toluene adsorbed by the adsorbent at equilibrium, *q*_*e*_*(mg/g)*, was calculated by using the following equation:

2 $$ {q}_e=\frac{V\left({C}_0-{C}_e\right)}{W} $$

Where *C*_*0*_ and *C*_*e*_ (*mg/L*) are the initial and equilibrium concentrations of toluene, respectively, *V* (*L*) is the volume of the toluene solution and *W* (*g*) is the adsorbent mass.

## Analysis

The residual toluene concentrations in aqueous medium were determined using a Perkin-Elmer spectrophotometer corresponding to maximum wavelength (*λ*_*max*_) of toluene. The XRD analysis was performed on natural and modified diatomite samples using a Philips Xpert x-ray diffractometer. Scanning electron microscopic (SEM) of both natural and modified diatomite were carried out using LEO 1455VP scanning electron microscope before and after modification process. The samples were coated with gold (Au) prior to the scanning in the electron microscope. By using nitrogen adsorption method the BET specific surface area adsorbents was measured, using Autosorb-*1MP* apparatus from Qantachrome at *77 K*. The analysis on compositions was determined by energy dispersive X-ray technique using EDX-System (Tescan Vega /2/ Instrument) which is fitted to the SEM instrument.

## Results and discussion

### Surface characterization

**Figure 1:**
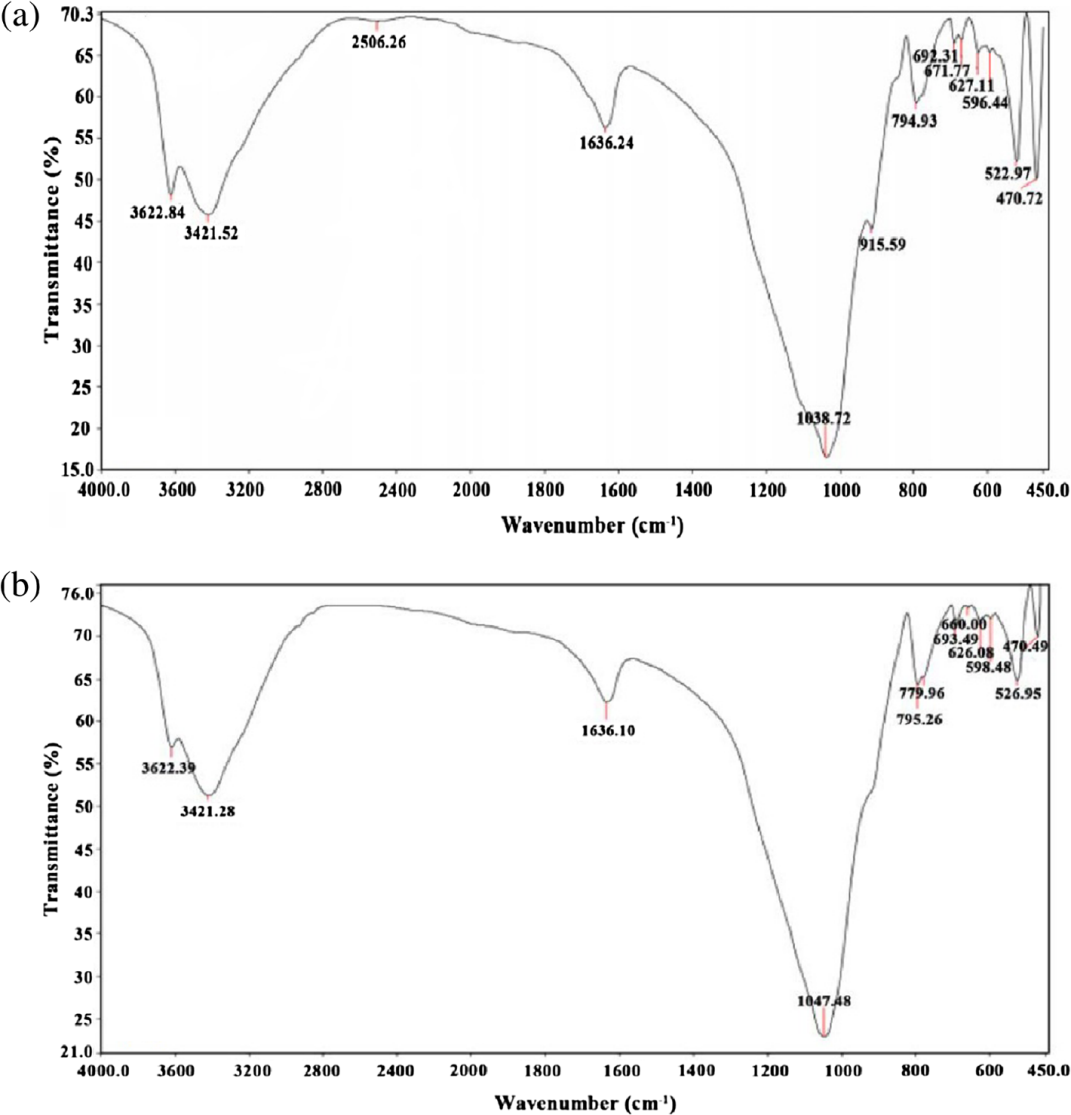
**FT-IR spectra of adsorbents before adsorption (a) natural and (b) NONMD.**

**Figure 2:**
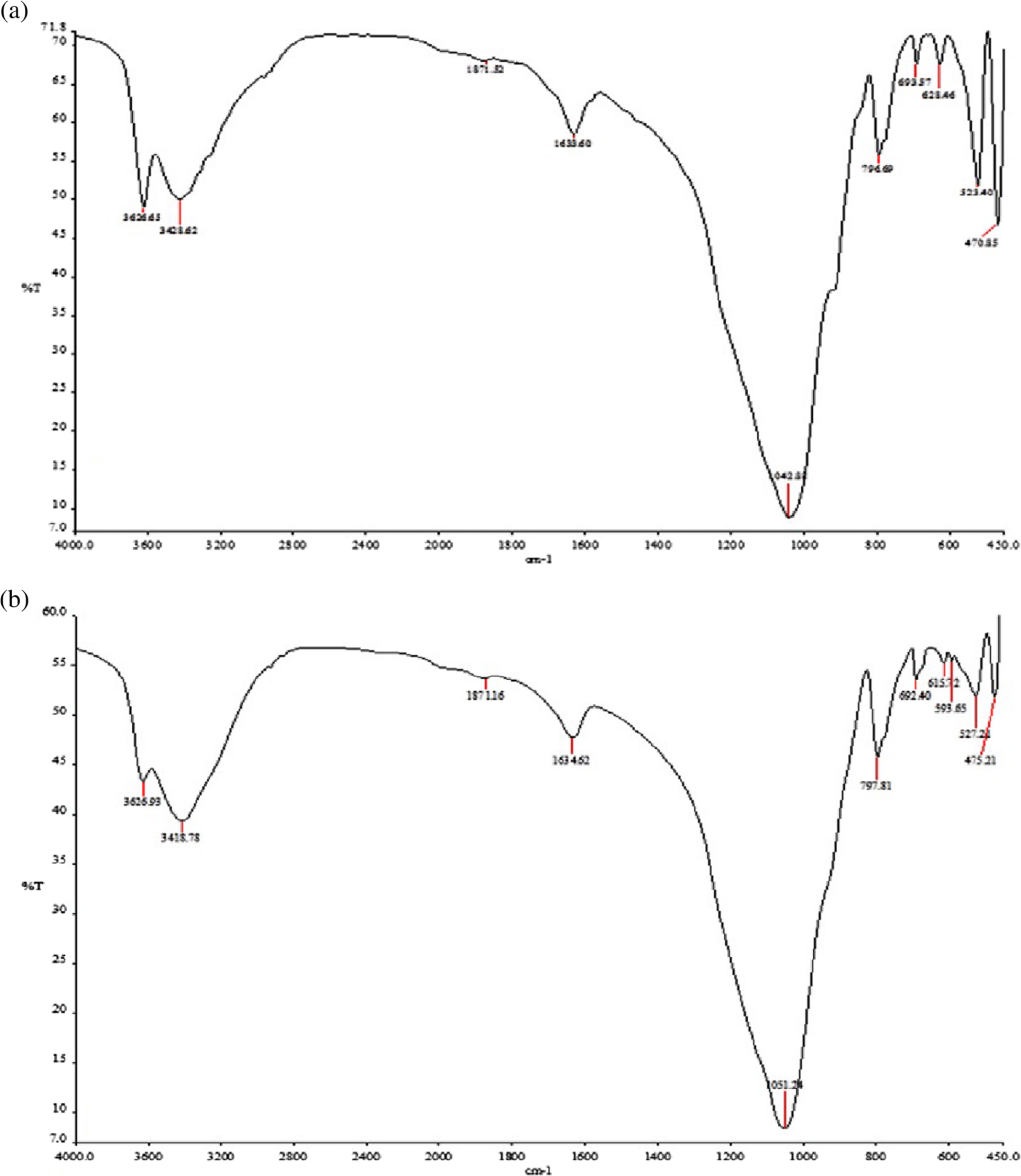
**FT-IR spectra of adsorbents after adsorption (a) natural and (b) NONMD.**

As the calcination process decreases the diatomite capacity for the removal of dye from aqueous solutions, therefore in this investigation, we decide to compare adsorption properties of modified diatomite with natural diatomite instead of modified diatomite. In order to explore the surface characteristics of diatomite, a Fourier Transformed Infra-Red (FTIR) analysis was performed in the range of *450* to *4000 cm*^*−1*^. Figure 1a and b show the FTIR spectra of natural and modified diatomite samples before the adsorption process, respectively. On the other hand, the FTIR spectra of both samples after the adsorption process are shown in Figure 2a and b. The FTIR spectral values and specific type of bonds are given in Table 1.

**Table 1:** **FTIR spectroscopy of spectra of natural and modified diatomite before and after the adsorption process**

**Steps**	**Adsorbents**	**Wavenumber (cm** ^**−1**^ **)**	**Specific type of bond**
Before the adsorption process	Natural diatomite	470, 522, 596, 627, 671, 692	Si-O-Si bending vibration
		794, 915	SiO-H vibration
		1038	Siloxane group stretching (-Si-O-Si-)
		1636	Bending vibration of water (H-O-H)
		2506	Silanol group (Si-O-H)
		3421, 3622	Si-H
Before the adsorption process	Modified diatomite (NONMD)	470, 526, 598, 626, 660, 693	Si-O-Si bending vibration
		779, 795	SiO-H vibration
		1047	Siloxane group stretching (-Si-O-Si-)
		1636	Bending vibration of water (H-O-H)
		2506	Silanol group (Si-O-H)
		3421, 3622	Si-H
After the adsorption process	Natural diatomite	470, 523, 628, 693	Si-O-Si bending vibration
		796, 915	SiO-H vibration
		1042	Siloxane group stretching (-Si-O-Si-)
		1633	Bending vibration of water (H-O-H)
		1871	Combi group of aromatic rings
		2506	Silanol group (Si-O-H)
		3421, 3622	Si-H
After the adsorption process	Modified diatomite (NONMD)	475, 527, 593, 615, 692	Si-O-Si bending vibration
		797	SiO-H vibration
		1051	Siloxane group stretching (-Si-O-Si-)
		1634	Bending vibration of water (H-O-H)
		1871	Combi group of aromatic rings
		2506	Silanol group (Si-O-H)
		3418, 3626	Si-H

As can be seen in Figure 1b, the nickel content of NONMD is too low and there is a recognisable difference in this region regarding to nickel oxide nanoparticles on diatomite [35]. In addition, Figure 1a and b illustrate that the amount of silanol group has decreased slightly, and the metal oxide content has risen at the modified diatomite; however, the adsorption capacity has increased at NONMD. Comparison of Figures 1 and 2 show that there is a new peak at *1871 cm*^*−1*^ for the adsorption of dye (combi group of aromatic rings) on both samples after the adsorption process and peaks’ transmittance of silanol, siloxane, and metal oxide groups is slightly decreased in Figure 2.

**Figure 3:**
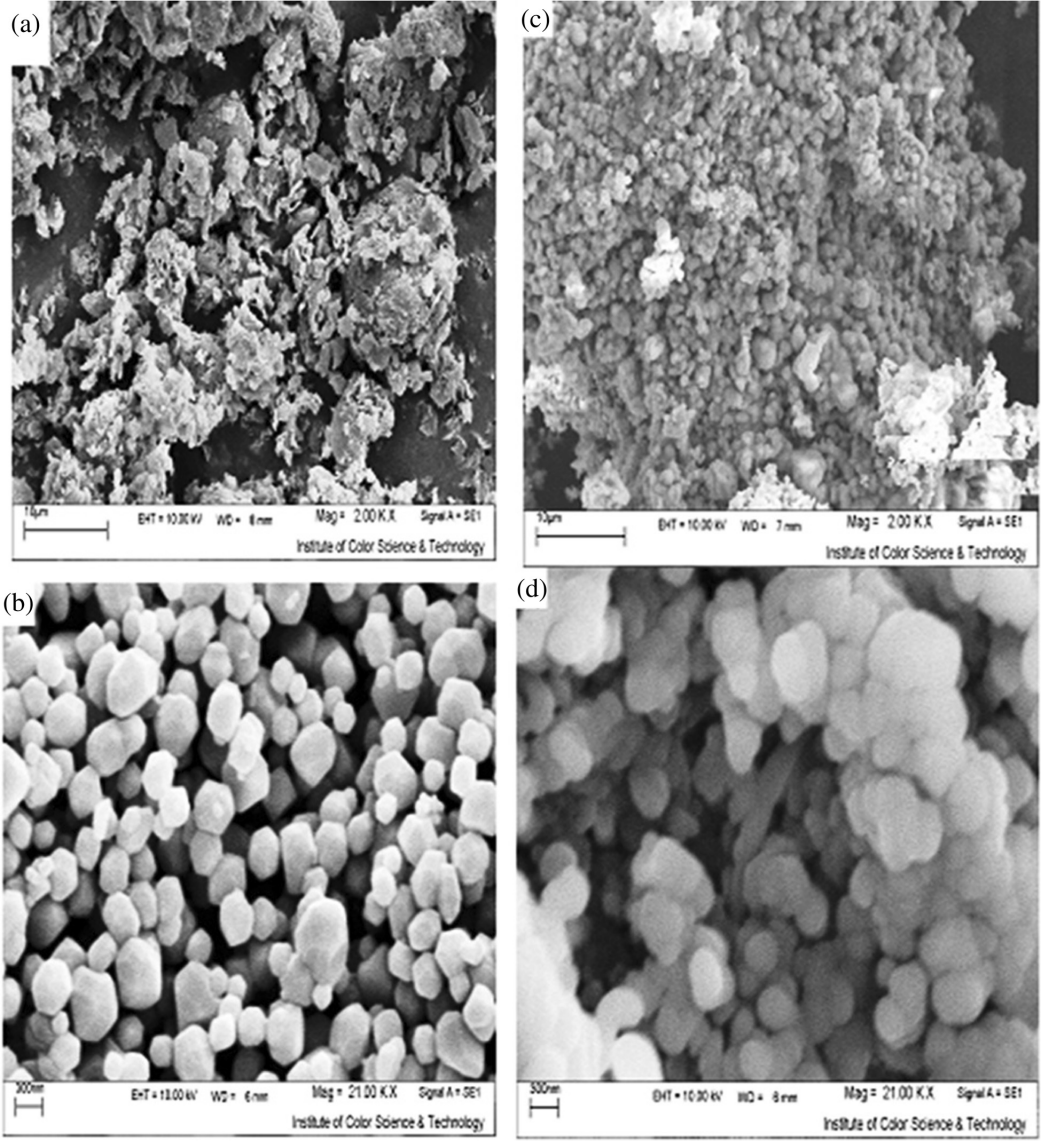
**Scanning electron micrographs before adsorption (a) natural and (b) NONMD, after adsorption (c) natural and (d) NONMD.**

Scanning electron micrographs (SEM) of natural and modified diatomite, before and after the adsorption process, are shown in Figure 3. These figures present that natural diatomite particles are amorphous, their pore spaces are lower than modified adsorbent, and particles of NONMD are more uniform and geometrical significantly. After modification and calcination process at *250°C*, the structure of the adsorbent changed from amorphous sheets to semi-sphere shapes. Therefore, the volume of the pore spaces has increased, and the surface functional groups of modified diatomite have improved. Hence, the diffusion resistance of adsorption process has decreased, and the kinetics and capacity of dye adsorption process have risen. Moreover, adsorption of dye has caused a sticky phenomenon on structure of both adsorbents.

**Figure 4:**
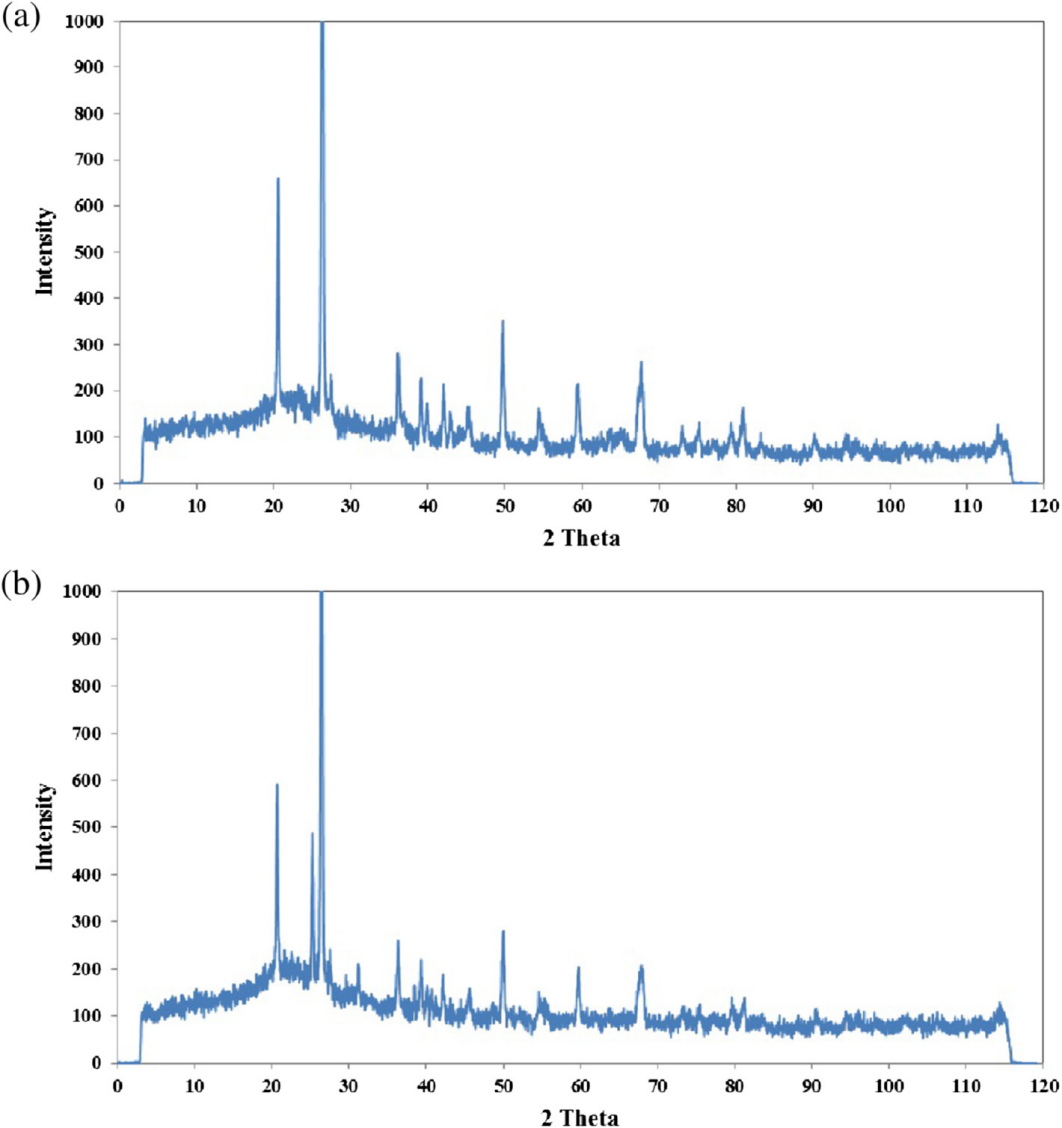
**XRD patterns of (a) natural and (b) modified diatomite.**

XRD analysis results of the natural and modified diatomite are shown in Figure 4. It can be seen from Figure 4 that the x-ray pattern of the natural diatomite is different from the pattern of the modified diatomite, suggesting that a phase transformation probably occurred during the modification and calcinations processes. The main composition of natural diatomite is quartz, anorthite and muscovite. It is evident that sanidine was appeared; while anorthite and muscovite were completely removed as the diatomite was calcined at *250°C*.

In fact, some peaks in the diatomite disappeared and some peaks were created by modification process. It can be a trace of nickel oxide particles particles from modification process and ammonia ions because of calcination at high temperature and existence of nitrogen in air. Similar behaviour was previously investigated by other researchers [19,35].

**Figure 5:**
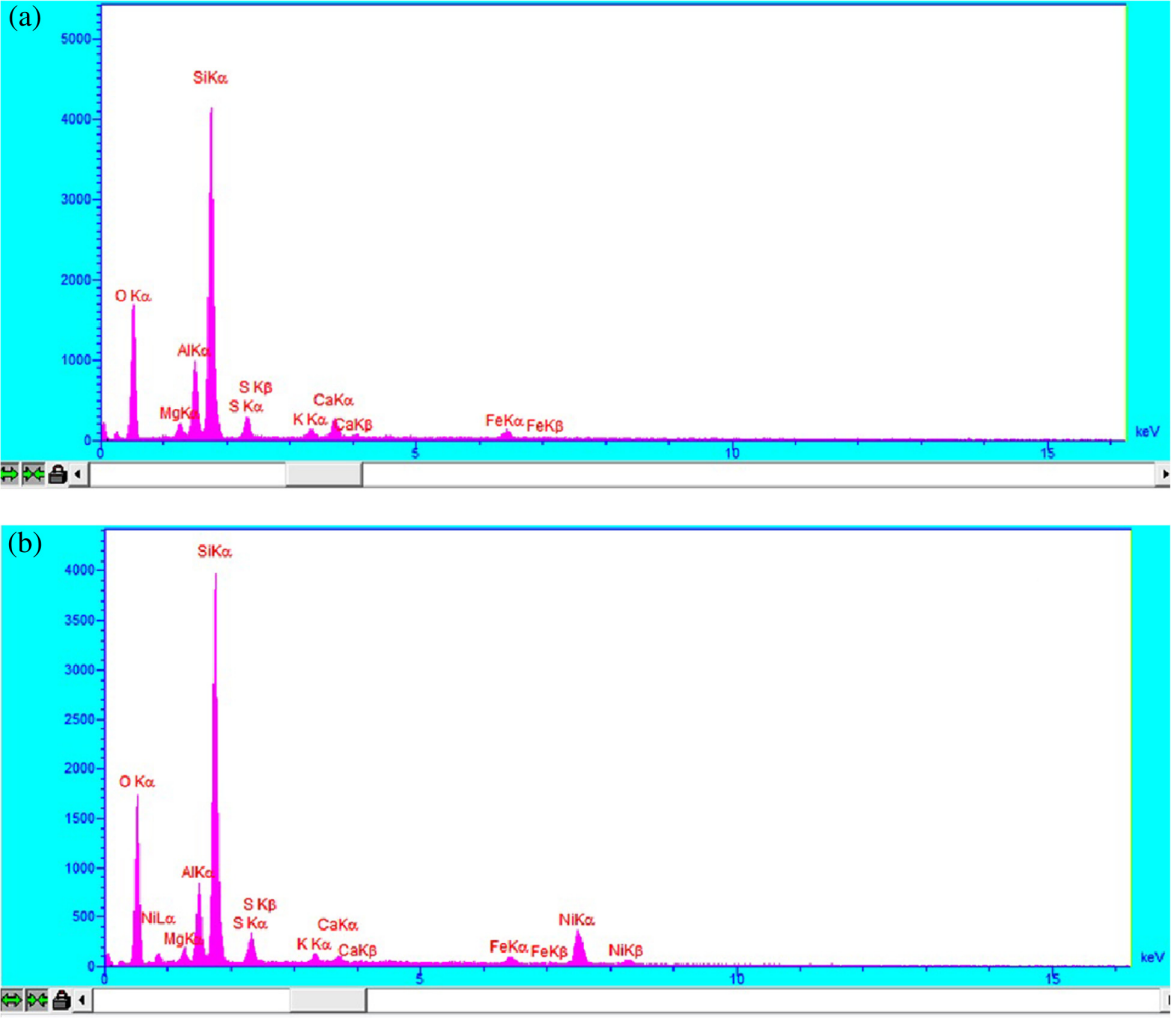
**EDX patterns of (a) natural and (b) modified diatomite.**

**Figure 6:**
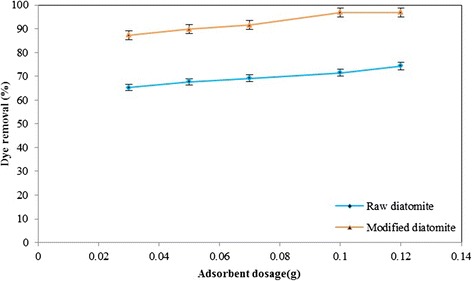
**Effect of adsorbent dosage on the removal percentage of toluene by natural and modified diatomite, Temperature =** ***25 ± 1°C*** **, initial toluene concentration =** ***150 mg/L*** **,** ***pH = 6*** **, agitation speed =** ***200 rpm*** **.**

As evident from EDX analyses (Figure 5), the natural diatomite did not have any Nickel but after modification process Ni was appeared (*3.96%W*). The surface area of the diatomite was determined by BET method (Figure 6). By using nitrogen adsorption method the BET specific surface area adsorbents was measured, using Autosorb-*1MP* apparatus from Qantachrome at *77 K*. In this investigation the values *7.5* (*m*^*2*^*/g*) and *28.45* (*m*^*2*^*/g*) for natural and modified diatomite were calculated respectively.

### Effect of adsorbent dosage

The effect of natural and modified diatomite dosage on the adsorption of toluene was investigated at *25 ± 1°C* by varying the adsorbent amount from *0.03* to *0.12 g* while keeping the volume of toluene solution constant equal to *100 mL*, with an initial toluene concentration of *150 mg/L*. Figure 6 shows the percentage removal of toluene versus adsorbent amount. It is clear from the Figure 6, the removal percentage of toluene increased with an increase in the adsorbent amount. The main reason for this fact is due to the greater availability of the adsorption sites at higher concentrations of the adsorbent [19,35-37]. Based on the results shown in Figure 6, *0.1 g* of the natural and modified diatomite was used for further experiments.

### Effect of initial toluene concentration

**Figure 7:**
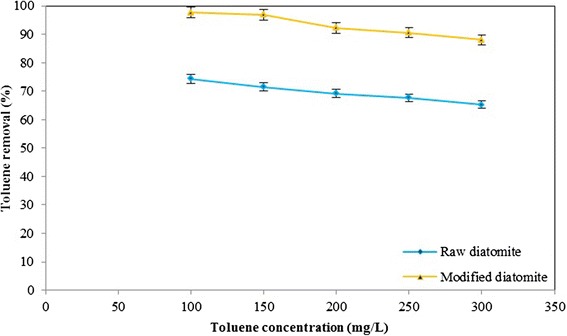
**Effect of initial toluene concentration on adsorption of toluene by natural and modified diatomite, Contact time =** ***90 min*** **, Temperature =** ***25 ± 1°C*** **,** ***pH = 6*** **, agitation speed =** ***200 rpm*** **.**

A change in the initial toluene concentration can considerably affect the adsorption process. Figure 7 depicts the effect of toluene concentration on the percentage removal of toluene by adsorbents. Evident from the Figure 7, when the toluene concentration increased from *100* to *300 mg/L* the percentage removal of toluene decreased from *97.68* to *88.09%* for modified and from *74.3* to *65.21* for natural diatomite.

As expected, when the concentration of toluene is increased, the limited capacity of the adsorbent checks any further adsorption of toluene and hence the overall removal percentage decreased.

### Effect of contact time

**Figure 8:**
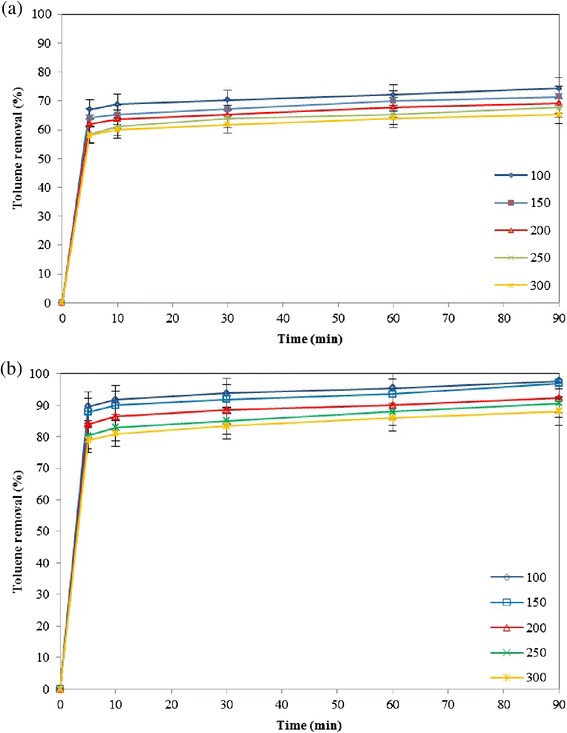
**Effect of contact time on adsorption of toluene on (a) natural and (b) modified diatomite, Equilibrium time =** ***90 min*** **,** ***pH = 6*** **, agitation speed =** ***200 rpm*** **, adsorbent dosage =** ***0.1 g*** **.**

The adsorption of toluene onto diatomite was evaluated as a function of contact time. Figure 8 shows the effect of contact time on the percentage removal of toluene in the aqueous phase by natural (Figure 8a) and modified diatomite (Figure 8b). The initial toluene concentration was varied from *100* to *300 mg/L*. At all initial toluene concentrations investigated, the adsorption occurs very fast initially. After *5 min* of adsorption process, the amount of adsorption by natural diatomite reaches to *66.99* and *64.09%* of the ultimate adsorption of toluene for initial toluene concentrations of *100* and *150 mg/L* respectively. As illustrated in Figure 8b, the adsorption is also fast at early stage of the adsorption process for modified diatomite. Typically about *87.87%* of the ultimate adsorption of toluene with an initial concentration of *150 mg/L* takes place within the first *5 min* of contact and it almost remains constant thereafter. It means that the most of mass transfer resistance is in bulk of fluid and high rate agitation would decrease this resistant. In addition, these results show that the most of the toluene molecules are adsorbed on the external surface of the adsorbent, and transferred to the pores and internal surfaces layer. More experiments are necessary to be carried out to prove this investigation. As expected, when the concentration of toluene is increased, the limited capacity of the adsorbent checks any further adsorption of toluene and hence the overall removal percentage decreases.

## Adsorption isotherms

**Figure 9:**
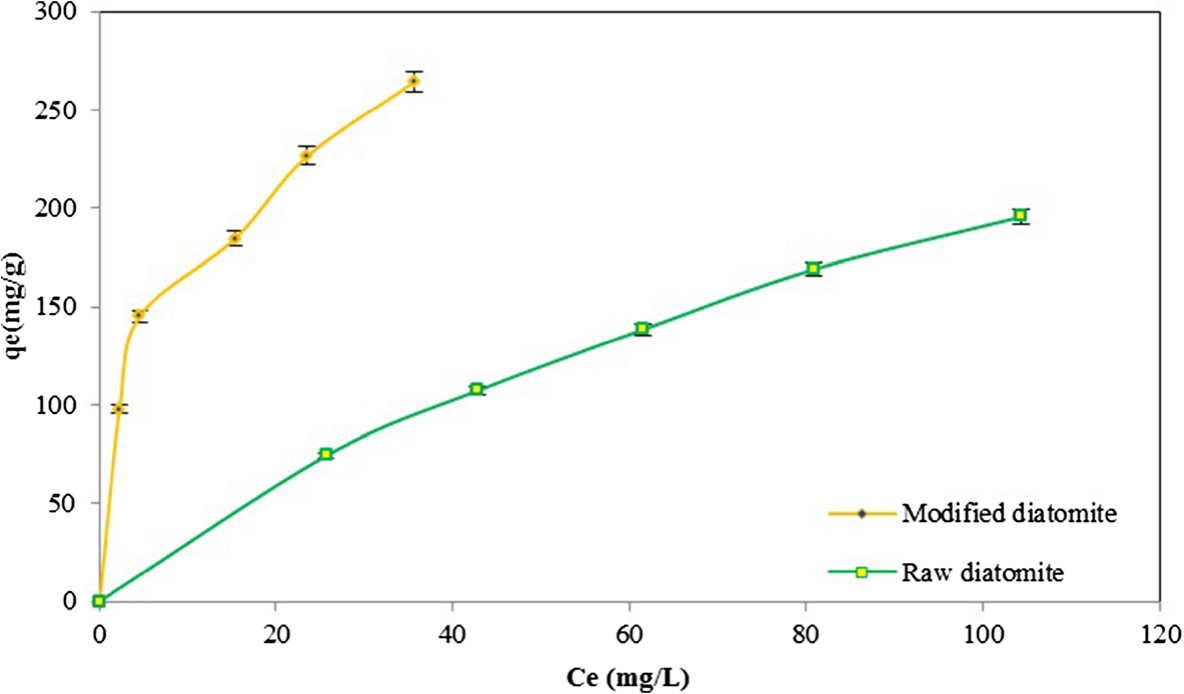
**Adsorption isotherms of toluene onto natural and modified diatomite.**

The distribution of toluene between the adsorbent and the toluene solution at equilibrium is important in establishing the capacity of the adsorbent for toluene removal from aqueous systems. The adsorption isotherms of toluene on both natural and modified diatomite are shown in Figure 9. It is clearly seen from Figure 9 showed that the amount of adsorbed toluene on natural diatomite was much lower than that of modified diatomite.

The experimental data obtained were evaluated by various isotherm models incorporating Langmuir, Freundlich [37-39] and Brunauer-Emmett-Teller (BET) [39-41] isotherms.

Langmuir isotherm is applicable for monolayer adsorption on a surface containing a finite number of identical adsorption sites [42,43]. A linear expression for the Langmuir isotherm is as follows:

3 $$ 1/{q}_e=\left(\frac{1}{K_L\kern0.1em {Q}_0}\right)\left(1/{C}_e\right)+1/{Q}_0 $$

Where *C*_*e*_ is the concentration of toluene under equilibrium condition (*mg/L*), *q*_*e*_ denotes the amount of toluene adsorbed at equilibrium (*mg/g*), *Q*_*0*_ indicates the maximum adsorption capacity and *K*_*L*_ is the Langmuir isotherm constant (*L/mg*). The values of *K*_*L*_ and *Q*_*0*_ were calculated from the slope and intercept of the linear plot of *1/q*_*e*_ versus *1/C*_*e*_.

Freundlich equation was also applied for the adsorption of toluene on diatomite as given below:

4 $$ {q}_e={K}_F\kern0.1em {C_e}^{\frac{1}{n}} $$

Where *C*_*e*_ is the equilibrium toluene concentration in aqueous system (*mg/L*), *q*_*e*_ is the amount of toluene adsorbed per weight of the adsorbent used (*mg/g*), *K*_*F*_ and n are Freundlich isotherm constants incorporating all factors affecting the adsorption process. Taking log_10_ from both sides of the Eq. (4) yields the following equation:

5 $$ \log {}_{10}\;{q}_e= \log {}_{10}\;{K}_F+\frac{1}{n\;}\; \log {}_{10}\;{C}_e $$

Linear plot of log_10_ *q*_*e*_ versus log_10_ *C*_*e*_ gives the values of *K*_*F*_ and *n*.

Brunauer-Emmett-Teller (BET) model was also used to fit the adsorption data according to the linear form of its rearranged adsorption isotherm model, which may be expressed as:

6 $$ \frac{C_e}{\left({C}_s-{C}_e\right)\kern0.1em {q}_e}\kern0.5em =\kern0.5em \frac{1}{K_b\;{q}_m}\kern0.5em +\kern0.5em \left(\frac{K_b-1}{K_b\kern0.1em {q}_m}\right)\;\left(\frac{C_e}{C_s}\right) $$

where *C*_*e*_ is the concentration of toluene in solution (*mg/L*), *C*_*s*_ denotes the saturation concentration of toluene (*mg/L*), *q*_*e*_ is the amount of toluene adsorbed per weight of the diatomite used (*mg/g*), *q*_*m*_ is the amount of toluene adsorbed in forming a complete monolayer (*mg/g*), *K*_*b*_ indicates a constant explaining the energy of interaction with the surface. The values of *K*_*b*_ and *q*_*m*_ were calculated from the slope and intercept of the linear plot of $\left(\frac{C_e}{C_S-{C}_e}\right)\;\frac{1}{q_e}\;\mathrm{versus}\;\frac{C_e}{C_S}.$

The *Q*_*0*_, *K*_*L*_, *r*_*1*_^*2*^ (correlation coefficient for Langmuir isotherm), *K*_*F*_, *1/n*, *r*_*2*_^*2*^ (correlation coefficient for Freundlich isotherm), *K*_*b*_, *q*_*m*_ and *r*_*3*_^*2*^ (correlation coefficient for BET isotherm) are given in Table 2. The negative values of *K*_*b*_ related to the BET isotherm model describe that the adsorption process for natural diatomite did not follow the BET isotherm model, since this constant is indicative of the surface binding energy. In addition, standard statistics of root mean squared error (RMSE) was carried out to support the best fit adsorption model. RMSE can be expressed as:

**Table 2:** **Parameters of various isotherms for adsorption of toluene onto natural diatomite and NONMD**

	**Langmuir**				**Freundlich**				**BET**			
**Adsorbent**	**Q**_**0**_	**K**_**L**_	**r**_**1**_^**2**^	**RMSE**	**K**_**F**_	**1/n**	**r**_**2**_^**2**^	**RMSE**	**K**_**b**_	**q**_**m**_	**r**_**3**_^**2**^	**RMSE**
**Natural**	263.150	0.008	0.997	0.250	7.760	0.697	0.970	0.410	0.873	1.922	0.819	0.490
**NONMD**	400.000	0.256	0.976	0.320	78.144	0.336	0.998	0.210	11.520	0.128	0.886	0.310

7 $$ RMSE={\left[\frac{1}{n}{\displaystyle \sum {\left({q}_p-{q}_O\right)}^2}\right]}^{\frac{1}{2}} $$

Where *q*_*p*_ is the predicted sorption capacity (*mg/g*), *q*_*o*_ is the observed sorption capacity (*mg/g*) and *n* is the number of samples. Thus, based on the high *r*^*2*^ and low RMSE values, the results present that the adsorption of BR 46 on the natural diatomite and the NONMD follow the Langmuir model and the BET model, respectively [27]. It is evident from Table 2 that the isotherm data for the adsorption of toluene by natural diatomite were best-fitted using Langmuir model with a correlation coefficient of *0.997*. Furthermore, the Freundlich model is most appropriate for the adsorption of toluene on modified diatomite with a correlation coefficient of *0.998*.

### Adsorption kinetics

The prediction of the adsorption kinetics of toluene from aqueous system is important in order to design a suitable treatment system. The kinetics of adsorption of toluene on diatomite may be described by the pseudo-first-order Lagergren rate equation [43-45] and the pseudo-second-order rate expression developed by Ho and McKay [46-49]. The Lagergren equation is:

8 $$ \log\;\left({q}_e-{q}_t\right)= \log \kern0.2em {q}_e-\frac{K_{1,\; ad}}{2.303}\kern0.22em t $$

Where *q*_*e*_ and *q*_*t*_ are the amounts of toluene (*mg/g*) adsorbed at equilibrium and at time *t* (*min*) and *K*_*1,ad*_ is the pseudo-first-order rate constant (*1/min*) [49].

The Ho and McKay equation is given below:

9 $$ \frac{t}{q_t}=\frac{1}{K_{2,\kern0.1em  ad}\kern0.2em {q_e}^2}+\frac{t}{q_e} $$

Where *q*_*e*_ and *q*_*t*_ are the amounts of toluene (*mg/g*) adsorbed at equilibrium and at time *t* (*min*) and *K*_*2,ad*_ is the rate constant of the pseudo-second -order model (*g/mg.min*) [49].

Linear plot of log_10_ (*q*_*e*_ − *q*_*t*_) against t gives the rate constant of *K*_*1,ad*_ Moreover, the value of *K*_*2,ad*_ is obtained from the intercept of the linear plot of *t/q*_*t*_ versus *t*. Adsorption kinetics constants of the pseudo-first-order and pseudo-second-order models at pH *6*, temperature *25 ± 1°C*, an agitation speed of 200 rpm, an initial concentration of 150 *mg/L* and for a time period of *90 min* are given in Table 3. From Table 3, the high values of correlation coefficients of the pseudo-second-order model for both natural and modified diatomite showed that the adsorption data conformed well to the Ho and McKay kinetics model [Eq. (8)].

**Table 3:** **Kinetics constants for toluene adsorption by natural diatomite and NONMD**

	**Pseudo-first-order**				**Pseudo-second-order**			
**Adsorbent**	**q**_**e**_ **(mg/g)**	**K**_**1,ad**_ **(1/min)**	**R** ^**2**^	**RMSE**	**q**_**e**_ **(mg/g)**	**K**_**2,ad**_ **(g/mg min)**	**R** ^**2**^	**RMSE**
**Natural**	49.653	0.654	0.875	0.121	123.451	0.018	0.991	0.114
**NONMD**	73.345	0.873	0.888	0.117	193.431	0.053	0.993	0.111

## Conclusions

Diatomite has been studied for the removal of toluene from aqueous solution. Modification treatment of the adsorbent with nickel oxide nanoparticles was useful and its adsorption capacity increased. The adsorption process was not influenced by solution pH and used natural pH of the solutions. It was found that in order to obtain the highest possible removal of toluene, the experiments can be carried out at pH *6*, temperature *25°C*, an agitation speed of *200 rpm*, an initial toluene concentration of *150 mg/L*, a centrifugal rate of *4000 rpm*, adsorbent dosage = *0.1 g* and a process time of *90 min*. The results of this work show that the maximum percentage removal of toluene from aqueous solution in the optimum conditions for NONMD was *96.91%* (*145.36 mg/g*). Furthermore, under same conditions, the maximum adsorption of natural diatomite was *71.45%* (*107.18 mg/g*).

The most important thing to design and run an industrial adsorption plant is the knowledge of adsorption kinetics and isotherms. Hence, the experimental results were analyzed by using the Langmuir, Freundlich and BET equations. Equilibrium data for the adsorption of toluene by natural diatomite fit well to the Langmuir isotherm model. Furthermore, the Freundlich model is most appropriate for the adsorption of toluene on NONMD. In addition, the rate of adsorption process for both of them obeys the pseudo-second-order kinetics model.

### Symbols used

*C*_*0*_ [*mg L*^*−1*^] initial concentration of toluene

*C*_*e*_ [*mg L*^*−1*^] residual concentration at equilibrium

*C*_*s*_ [*mg L*^*−1*^] saturation concentration of toluene

*K*_*1,ad*_ [*min*^*−1*^] rate constant of Pseudo-first-order

*K*_*2,ad*_ [*min*^*−1*^] rate constant of Pseudo-second-order

*k*_*F*_ [*(mg g*^*−1*^*)( mg L*^*−1*^*)*^*-1/n*^] Freundlich constant

*k*_*L*_ [*L mg*^*−1*^] Langmuir constant

*k*_*b*_ [*–*] BET constant

*m* [*g*] mass of adsorbent used

*n* [*–*] parameter indicating the intensity of adsorption

*q*_*e*_ [*mg g*^*−1*^] residual amount adsorbed at equilibrium

*q*_*p*_ [*mg g*^*−1*^] predicted sorption capacity

*q*_*o*_ [*mg g*^*−1*^] observed sorption capacity

*Q*_*o*_ [*mg g*^*−1*^] maximum sorption capacity

*r*^*2*^ [–] correlative coefficient for all models

*V* [*L*] volume of toluene solution
